# Understanding and Designing a High-Performance Ultrafiltration Membrane Using Machine Learning

**DOI:** 10.1021/acs.est.2c05404

**Published:** 2023-02-15

**Authors:** Haiping Gao, Shifa Zhong, Raghav Dangayach, Yongsheng Chen

**Affiliations:** † School of Civil and Environmental Engineering, Georgia Institute of Technology, Atlanta, Georgia 30332, United States; ‡ School of Ecological and Environmental Sciences, East China Normal University, Shanghai 200241, China; ∥ Shandong Provincial Key Laboratory of Water Pollution Control and Resource Reuse, School of Environmental Science and Engineering, Shandong University, Qingdao, Shandong 266237, China

**Keywords:** ultrafiltration membrane, machine learning, antifouling potential, water permeability, membrane properties

## Abstract

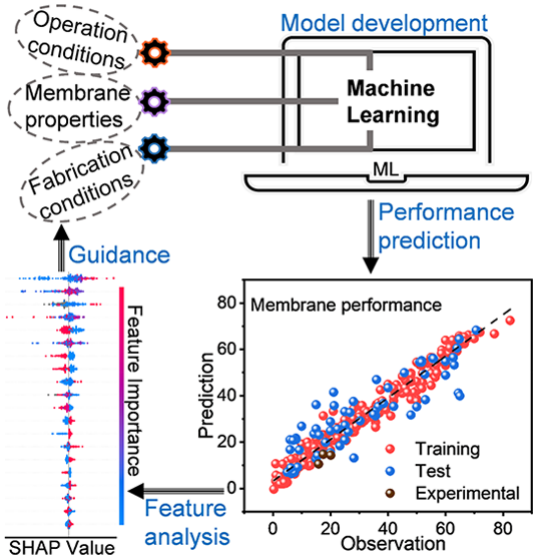

Ultrafiltration (UF) as one of the mainstream membrane-based technologies has been widely used in water and wastewater treatment. Increasing demand for clean and safe water requires the rational design of UF membranes with antifouling potential, while maintaining high water permeability and removal efficiency. This work employed a machine learning (ML) method to establish and understand the correlation of five membrane performance indices as well as three major performance-determining membrane properties with membrane fabrication conditions. The loading of additives, specifically nanomaterials (*A*_wt %), at loading amounts of >1.0 wt % was found to be the most significant feature affecting all of the membrane performance indices. The polymer content (*P*_wt %), molecular weight of the pore maker (*M*_Da), and pore maker content (*M*_wt %) also made considerable contributions to predicting membrane performance. Notably, *M*_Da was more important than *M*_wt % for predicting membrane performance. The feature analysis of ML models in terms of membrane properties (i.e., mean pore size, overall porosity, and contact angle) provided an unequivocal explanation of the effects of fabrication conditions on membrane performance. Our approach can provide practical aid in guiding the design of fit-for-purpose separation membranes through data-driven virtual experiments.

## Abstract 2

This study demonstrates the application of a machine learning method in deciphering the fabrication−property−performance relationship of polymeric membranes, offering new insights into the development of separation membranes with desirable performance in practical water treatment applications.

## Introduction

One of the most prominent issues of our time is the increasing supply–demand gap of clean and safe water due to industrialization, population growth, and global climate change. There is a critical need to develop sustainable water treatment technologies. In this context, membrane technologies such as reverse osmosis (RO), nanofiltration (NF), and ultrafiltration (UF) have emerged as promising alternatives to traditional water treatment practices owing to their compelling advantages such as high energy efficiency and fewer chemical additives.^1,2^ Of these, UF has been recognized as one of the mainstream membrane-based separation technologies in water and wastewater treatment applications, including the pretreatment stage for RO processes, membrane bioreactors, and water reclamation of effluent from wastewater treatment plants (WWTPs).^3−5^

At the core of the UF separation process is the UF membrane capable of rejecting large molecules [e.g., natural organic matter (NOM)] and bacteria from impaired water sources to produce a clean permeate. UF membranes can also be tuned to target the removal of specific compounds such as heavy metals and dyes, which makes it an attractive candidate for the treatment of industrial effluents.^6−11^ In the past several decades, there has been a rapid growth in UF membrane fabrication, including the exploration of membrane materials and the development of fabrication approaches (e.g., phase inversion, electrospinning, etc.).^12,13^ Notable examples include the emergence of adsorptive membranes^14,15^ and mixed matrix membranes (MMM) involving the incorporation of additives (e.g., nanomaterials) into the membrane matrix.^16−19^ However, due to the existence of various organic compounds (e.g., NOM and polysaccharides) and pathogenic microorganisms in water streams, membrane fouling has always been a major obstacle hampering the more widespread application of UF membranes.

Generally, membrane fouling can be classified as reversible fouling and irreversible fouling.^20−22^ The impaired membrane performance caused by reversible fouling can be recovered through hydraulic backwashing. However, irreversible fouling induced by the foulants bonded to membrane surface and trapped in pores can be eliminated only by chemical cleaning.^23^ Membrane fouling not only causes significant loss of water permeation and a shorter membrane lifetime but also increases operational cost and complexity.^24,25^ Therefore, fabricating UF membranes with desirable water permeance and removal efficiency accompanied by high antifouling potential is crucial. A polymeric membrane fabricated through a phase inversion method has been recognized as the state-of-the-art UF membrane. However, most polymeric membranes were fabricated using hydrophobic polymers such as polysulfone (PSf), poly(ether sulfone) (PES), and polyvinylidene fluoride (PVDF), suffering from poor water flux and membrane fouling. Many efforts, including developing block polymers, surface grafting, blending with hydrophilic polymers, and incorporation of inorganic fillers, have been devoted to developing high-performance UF membranes.^26−32^ One popular approach is to fabricate nanocomposite UF membranes by embedding nanomaterials into a polymer matrix. A wide variety of nanomaterials with different physical and chemical properties, ranging from nonporous nanoparticles (e.g., TiO_2_) and porous nanomaterials (e.g., carbon nanotubes) to two-dimensional (2D) materials (e.g., graphene oxide), have been investigated.^33−35^ With so much interest in nanocomposite UF membranes, identifying the desirable loading and properties of the nanomaterials as well as the suitable membrane fabrication conditions to optimize membrane performance is essential. As membrane performance is mostly determined by membrane properties such as surface hydrophilicity, surface roughness, effective pore size, and porosity, the correlation of membrane properties with fabrication conditions is also worth examining.

Due to the variety of membrane backbone materials and available additives as well as the complexity of the fabrication process, rationally designing UF membranes by developing efficient methods to alleviate the time and resource constraints posed by the iterative trial-and-error approach is pivotal. Because of its powerful ability in processing and learning from large, complex, and multidimensional data sets to develop predictive models, machine learning (ML) as a data-driven method has become increasingly important in chemistry and material science communities for accelerating the discovery of new materials and chemical synthesis.^36−40^ In most recent years, ML has been employed to guide gas separation membrane design.^41−43^ In addition, ML models such as tree-based models (e.g., random forest, XGBoost, etc.) and artificial neural networks (ANNs) have been developed to predict permeance and rejection for RO and NF membranes in water treatment and resource recovery, including but not limited to solvent recovery.^44−48^ Several studies have been acknowledged for the application of ML models in ML-assisted UF membrane fabrication.^49,50^ However, the performance of these reported models was often limited by the incomplete input variables and unclear classification of the input features. Furthermore, previous work has mainly focused on prediction of water flux and removal efficiency. Limited attention has been paid to predicting membrane fouling-related performance comprising the flux decline ratio, flux recovery ratio, reversible fouling ratio, and irreversible fouling ratio. Additionally, the quantitative relationship between fabrication conditions and membrane performance-determining properties has not been well established.

In this work, we developed tree-based ML models using extreme gradient boosting (XGBoost) and categorical boosting (CatBoost) as potential candidates to analyze a data set containing input features associated with both fabrication and operational conditions and to target membrane performance. As for membrane performance, we considered water permeability, removal efficiency, and indices associated with membrane antifouling performance, such as the flux decline ratio, flux recovery ratio, and reversible fouling ratio. The relative importance and impact of each feature on the target were evaluated using the Shapley additive explanations (SHAP) method to provide guidance on fabricating UF membranes with desirable water permeability, removal efficiency, and antifouling potential. Moreover, we also developed predictive ML models by correlating fabrication conditions with membrane properties and carried out model interpretations with the SHAP method. This interpretation facilitated a better understanding of the underneath mechanics in terms of the influence and contribution of each fabrication parameter to membrane performance. This work demonstrated the potential of ML methods in providing guidance to the fit-for-purpose membrane development to meet challenges in water and wastewater treatment and resource recovery.

## Data Set and Methods

### Data Collection and Data Set Construction

The quantity and quality of collected data used to develop ML models are crucial to the model prediction performance. To develop ML models with accurate prediction, we mined data from research articles associated with flat-sheet polymeric UF membranes fabricated by the most used non-solvent-induced phase separation (NIPS) method at room temperature to construct the data sets with the total size of 320. The full data sets can be found in the Supporting Information. Parameters and descriptors used as input features were exhaustively collected from tables, text, and graphical data. As summarized in Table S1, on the basis of the empirical domain knowledge, these input features were assembled into three categories consisting of fabrication conditions (11 variables), operational conditions (six variables), and membrane properties (four variables). The ratio between variables and total data size was calculated to be 6.6%. Features describing the fabrication and operational conditions involved both numerical parameters such as polymer concentration (*P*_wt %), pore maker content (*M*_wt %), and the loading of the additives (*A*_wt %) and categorical ones such as the types of pore makers and organic solvents (as summarized in Table S1). The distribution of the data of the numerical features is illustrated in Figure S1. In the constructed data sets, the additives were specifically referred to various types of nanomaterials (as categorized in Figure S2). The categorical features were first converted into numeric ones by the encoding methods. We screened eight encoding methods (as listed in Table S2) and selected the optimum one to convert these categorical features into numeric features. Notably, features with insufficient information such as the absence of molecular weights for polymers and pore makers were designated as missing values rather than being excluded. The base polymers were represented by molecular fingerprints encoding the repeating unit as a binary vector (0, 1) by converting its SMILES in Python’s RDKit package. The obtained molecular fingerprints of the polymers combined with other numerical features (including those from converted categorical features) were used as the final input features to develop ML models. Generally, three data sets were compiled, one for the prediction of membrane performance from fabrication and operational conditions, one for the prediction of membrane performance with the combination of fabrication/operational conditions and membrane properties as input, and the third for the prediction of membrane properties from fabrication conditions.

### Machine Learning Model Development and Evaluation

As all of the features have their specific physicochemical meanings, the missing values present in the input features were not imputed. The percentages of missing values for each feature are listed in Table S3. Hence, it is necessary to employ ML algorithms with the capability of processing missing values. Tree-based algorithms have been reported to exbibit satisfactory performance in handling missing values containing data sets. Here, we utilized two tree-based ML algorithms, i.e., XGBoost and CatBoost, as candidates. The tree-based ML model for each data set was developed in a similar manner with some modifications depending on the specific data set. Typically, the data set was randomly split into two parts: 80% of the whole data set as the training set and the remaining 20% of the data set as the test set for model evaluation. Five-fold cross-validation was employed on the training set to screen the ML algorithms, encoding methods, and hyperparameter tuning. After the optimum configuration of ML algorithms and/or encoding methods had been screened (Table S4), the hyperparameters of the ML algorithm were then tuned by using the Bayesian optimization algorithm (Table S5). The length and radius of the molecular fingerprint were considered as hyperparameters, which were tuned together with other hyperparameters of ML algorithms. After obtaining the optimum hyperparameters, we retrained the ML algorithm with the optimal hyperparameters on the whole training set (without using 5-fold cross-validation) to deliver the final ML models. The predictive performance (i.e., generalization ability) of ML models was evaluated on the test set. The coefficient of determination (*R*^2^) and root-mean-square error (RMSE) were utilized to evaluate the prediction accuracy as defined by eqs 1 and 2. The lower RMSE and higher *R*^2^ indicate the better predictive performance of ML models.
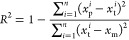
1
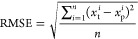
2where *x*_p_^*i*^ is the predicted value of the output, *x*_t_^*i*^ is the actual value of the output reported in the literature, *x*_m_ is the mean value of all of the output, and *n* is the number of data points in the training or test set.

### Feature Analysis

To understand the built models and thus provide insightful guidance for future membrane fabrication, identifying potential controlling fabrication parameters for membrane properties and performance is essential. In previous work, feature importance analysis has been performed to explain different models (such as random forest and neural network), providing insight into the roles of input features.^49,51^ Here, the importance and impact of each feature on the targets were analyzed using the SHAP method.^48^ The Shapley value for input feature *x* (of *n* total input features) given the prediction *p* by the built ML model was calculated by^52^
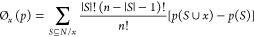
3where *S* is the subset for each feature without feature *x*, *p*(*S* ∪ *x*) represents the predictions by the built ML model considering feature *x*, and *p*(*S*) represents the predictions without considering feature *x*. The differences among all possible subsets of *S* ⊆ *n* are calculated due to the dependency of the effect of withholding a feature from other features in the ML model.

### Characterization of Membrane Properties

To further validate the model prediction accuracy, three UF membranes were fabricated using NIPS methods as described in Texts S1 and S2 and Table S6. The water contact angle (CA) indicating membrane surface hydrophilicity was measured by a Ramé-Hart model 250 goniometer (Ramé-Hart Instrument Co.). The average membrane pore radius (micrometers) was determined on the basis of the Guerout–Elford–Ferry equation^53^ as follows:
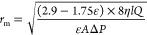
4where η is the water viscosity at 23 °C (9.3 × 10^–4^ Pa s), *Q* is the permeate flow rate (cubic meters per second), and Δ*P* is the operational pressure (pascals).

The overall porosity (percent) of a membrane was measured by the dry–wet weight gravimetric method as expressed by the following equation:
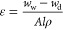
5where *w*_w_ is the weight of the hydrated membrane (grams), *w*_d_ is the weight of the dried membrane (grams), *A* is the surface area of the membrane (square centimeters), *l* is the membrane thickness (centimeters) determined by the cross-section SEM image, and ρ is the water density at 23 °C (0.998 g cm^–3^).

### Evaluation of Membrane Performance

The pure water permeability of the fabricated membranes was measured using a dead-end ultrafiltration cell (Amicon stirred cell, Millipore Sigma) with an effective membrane area of 13.4 cm^2^. The membranes were precompacted under 4 bar for 2 h before switching to the operating pressure of 1–1.5 bar. A bovine serum albumin (BSA) solution was used as the feed solution to test the rejection performance of the membranes. The operational conditions for each of the fabricated membranes are listed in Table S6. The concentration changes of BSA were determined using a Shimadzu TOC-L analyzer (Shimadzu Scientific Instruments). The water permeability (*A*) and rejection efficiency (*R*) were calculated using eqs 6 and 7.
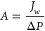
6
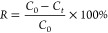
7where *J*_w_ is the water flux, Δ*P* is the applied hydraulic pressure, and *C*_0_ and *C*_*t*_ refer to the concentrations of BSA in the feed solution and permeate, respectively.

Membrane fouling performance was evaluated using humic acid (HA) as the model foulant. The membranes under investigation were first compacted for 2 h under applied hydraulic pressure to reach a steady water flux. The water flux was recorded for an additional 1 h to obtain the pure water flux (*J*_w_). Subsequently, the pure water was converted into the HA solution and allowed to run for 6 h at the same applied hydraulic pressure. The steady flux with a 20 mg L^–1^ HA solution as the feed was recorded as *J*_t_. Then, the fouled membrane was physically cleaned with deionized (DI) water. After physical cleaning, the recovered pure water flux (*J*_r_) was recorded for an additional 1 h with DI water as the feed solution. The flux decline ratio (FDR) coupled with the water flux recovery ratio (FRR) was used to evaluate the membrane antifouling potential based on eqs 8 and 9.^54^ Generally, the lower FDR and the higher FRR suggest a better antifouling performance of the membranes.
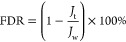
8
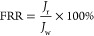
9

Membrane fouling can be generally classified as reversible and irreversible fouling. The flux decline ratio caused by reversible and irreversible fouling during the filtration process was calculated by
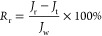
10
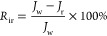
11

## Results and Discussion

### Predictive ML Models

To simplify the ML model and enhance its performance, the Pearson correlation coefficient (PCC) was determined to identify the correlations between features. Figure S3 shows the PCC results of all of the features. With respect to membrane performance, the irreversible fouling ratio showed a completely negative correlation with the flux recovery ratio (i.e., PCC = −1), which is in line with the calculation result according to eqs 9 and 11. Therefore, the irreversible fouling ratio was sorted out from the output target and the flux recovery ratio was chosen as the representative one.

Overfitting is controlled by tunning the hyperparameters of the machine learning algorithm to control the complexity of the model. We used the Bayesian optimization algorithm to tune the hyperparameters of the machine learning algorithm, in which we observed the training and validation performance together. We took the hyperparameters that can afford the best validation performance as the optimum hyperparameters. Figure S4 demonstrates the plot of training RMSE (RMSEcv-training) versus validation RMSE (RMSEcv-validation), in which the black dotted line indicated the lowest RMSEcv-validation. With a decrease in RMSEcv-training, RMSEcv-validation first decreased and then increased, indicating the underfitting and overfitting process. The location of the lowest RMSEcv-validation indicates the optimum complexity of the model.

**Figure 1:**
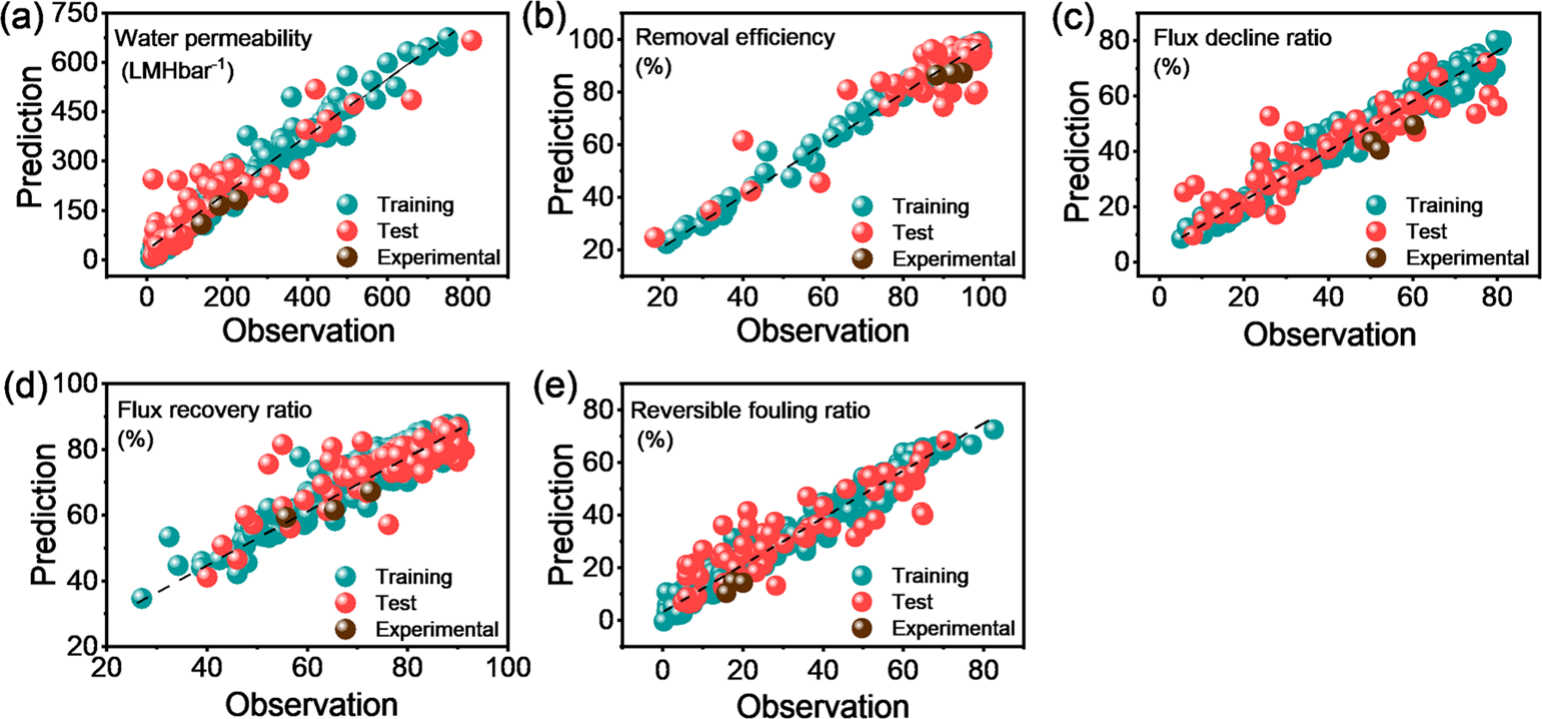
Prediction results of the ML models for each target: (a) water permeability, (b) removal efficiency, (c) flux decline ratio, (d) flux recovery ratio, and (e) reversible fouling ratio.

The predictive performance of the built ML model for each target is listed in Table 1 and plotted in Figure 1. The prediction of ML models in the test data sets exhibited an *R*^2^ value of ≥0.78 for water permeability, removal efficiency, and flux decline ratio. These results indicated the strongly quantitative correlation of membrane performance with the fabrication conditions and operational conditions. In comparison with the prediction performance of these three targets, the models exhibited relatively lower testing *R*^2^ values of 0.62 and 0.73 for the flux recovery ratio and reversible fouling ratio, respectively. As expressed in eqs 9 and 10, these two targets were highly relevant to the recovered water flux after physical cleaning. Therefore, the relatively limited prediction performance for the flux recovery ratio and reversible fouling ratio may be attributable to the case-by-case variations in the physical cleaning procedures. Improvements in the prediction performance could be achieved by expanding the current data sets to include more reasonable variables as input features such as the cleaning time of the physical cleaning process.

**Table 1:** Prediction Performance of the ML Models for Membrane Performance

performance index	total data size	training set *R*^2^	training set RMSE	test set *R*^2^	test set RMSE
water permeability (LMH bar^–1^)	320	0.97	29.61	0.83	68.24
removal efficiency (%)	320	0.99	1.83	0.84	6.60
flux decline ratio (%)	320	0.96	3.82	0.78	9.50
flux recovery ratio (%)	320	0.87	4.73	0.62	7.89
reversible fouling ratio (%)	320	0.96	3.91	0.73	10.33

As membrane performance mainly depends on membrane properties, we also developed ML models for predicting membrane performance by including membrane properties (e.g., mean pore radius, overall porosity, contact angle, and surface roughness) together with fabrication/operational conditions as input features. As listed in Table S7, the prediction of the ML models with membrane properties exhibited an *R*^2^ value comparable to those of the ML models without membrane properties. These results revealed the quantitative relationship among fabrication, property, and performance.

To further validate the generalization ability of the developed ML models, we fabricated three UF membranes (as listed in Table S6) and tested their performance (Table S8). Our experimental data points were independent of the 320 data points collected from the literature. We found that the experimental results for all of the targets showed relatively good agreement with the predicted values (Figure 1). This finding indicated that all of the built ML models were reliable enough to provide satisfactory predictions of membrane performance based on the selected input features. Notably, the polymers used to fabricate these three membranes have molecular weights different from those of the polymers enclosed in the collected data sets. The application of the molecular fingerprint method made it possible to deliver acceptable performance predictions for membranes fabricated with new polymers. However, the additive as a categorical feature was encoded to the numerical feature in this work, which limited the prediction capability of the built model for cases with new additives (i.e., those not present in the training data set).

**Figure 2:**
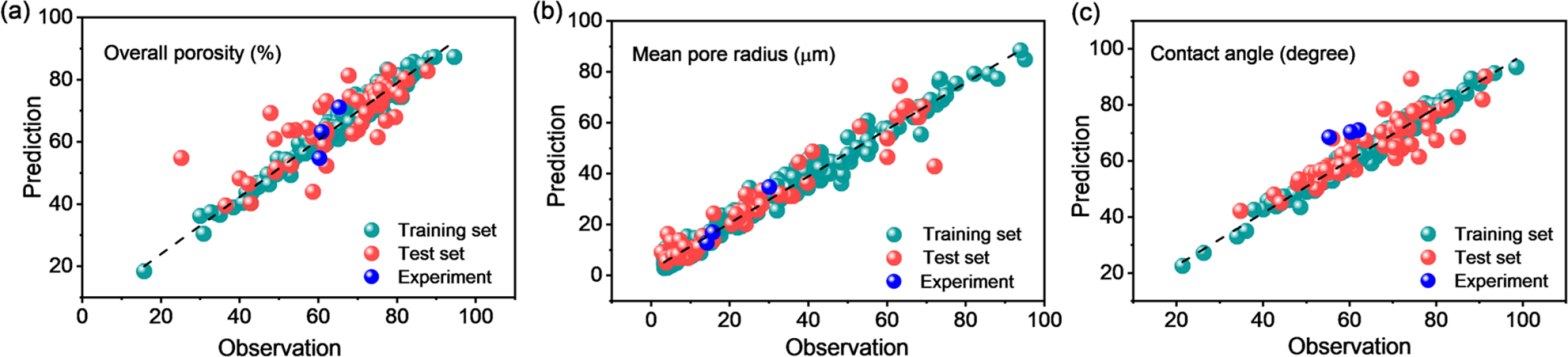
Prediction performance of the ML model for each membrane property: (a) prediction performance with experimental validation of the overall porosity, (b) prediction performance with experimental validation of the mean pore radius, and (c) prediction performance with experimental validation of the contact angle.

To understand the correlation between membrane properties and fabrication conditions, we also developed ML models to predict three major membrane performance-determining properties, i.e., mean pore radius, overall porosity, and contact angle (revealing the hydrophilicity of the membrane surface), using fabrication conditions as input features. As shown in Table 2, for the test data sets, the *R*^2^ values were 0.87 and 0.76 on the prediction of mean pore radius and contact angle, respectively, and their corresponding RMSEs were 7.59 and 5.87, respectively, suggesting satisfactory prediction performance of the built ML models with fabrication conditions as input features. In comparison with the prediction performance on mean pore radius and cotact angle, the *R*^2^ value of 0.66 for overall porosity was relatively lower, which could be partially explained by the variations in data quality due to the dry–wet gravimetric measurement method as calculated in eq 5. Notably, the observed membrane properties of our fabricated membranes (as listed in Table S9) were in line with the predicted values as indicated by the blue dots in Figure 2. This experimental validation further confirmed the prediction accuracy of the three predictive ML models on membrane properties.

**Table 2:** Prediction Performance of the ML Models for Membrane Properties

membrane property	total data size	training set *R*^2^	training set RMSE	test set *R*^2^	test set RMSE
overall porosity (%)	320	0.96	2.51	0.66	7.61
mean pore radius (μm)	320	0.98	3.47	0.87	7.59
contact angle (deg)	320	0.98	1.87	0.76	5.87

### Feature Analysis using the SHAP Method

**Figure 3:**
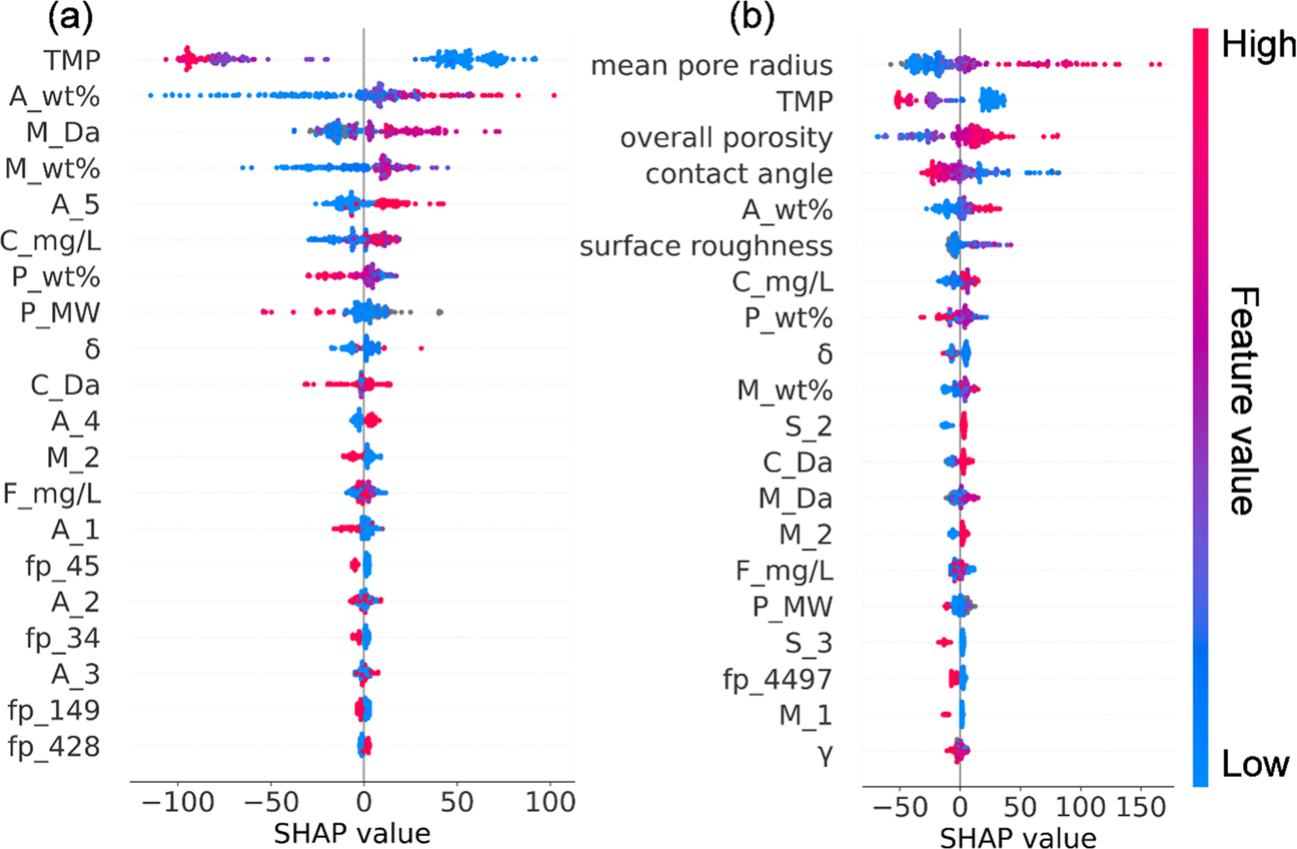
SHAP plot for water permeability based on ML models (a) without membrane properties and (b) with membrane properties. For panel a, the model was developed by using fabrication conditions as input features and water permeability as the target, while for panel b, the model was developed by using fabrication conditions combined with membrane properties as input features and water permeability as the target. *A*_number (e.g., *A*_5) denotes the encoder for the category feature (i.e., the type of additive). The feature number (e.g., fp_45) stands for the feature position in the Morgan fingerprint vector.

According to the built ML models, the contributions of each input feature to the targets (i.e., membrane performance) were evaluated using the SHAP method. A feature’s Shapley value quantifies its contribution, whether negative or positive. A feature with a higher absolute Shapley value implies a greater contribution to membrane performance. Figure 3a and Figure S5 illustrate the importance and impact of each feature on membrane performance. In general, the loading of additives (*A*_wt %), the polymer content of the total casting solution (*P*_wt %), the molecular weight of the pore maker (*M*_Da), and the pore maker content (*M*_wt %) were found to be the four most influential fabrication parameters for predicting membrane performance. Notably, *A*_wt % was found to be the most important fabrication parameter for predicting membrane performance indices except for the flux decline ratio, for which *A*_wt % ranked second in importance. With respect to water permeability as shown in Figure 3a, it is noteworthy that the feature *A*_5 also played an important role in improving water permeability. As described in the data collection and data set construction section, the category features were first converted into numerical features. Here, *A*_number stands for the encoder of the category feature (i.e., the type of additive). The SHAP plot demonstrated that *A*_5 positively contributed to water permeability, which means that the additive having an *A*_5 value of 1 (such as UiO-66) might be a desirable additive for enhancing water permeability. Because the additive was identified as a significant contributor to membrane performance, to optimize the robustness of the ML models and gain more insights into the effects of the additive, future work may focus on compiling data sets with structural parameters of the additives, especially nanomaterials (such as size, length, shape, and diameter) as well as its chemical properties (e.g., ζ potential, hydrophilicity, and surface functional groups).

Additionally, the operational conditions played considerably important roles in membrane performance. In particular, the transmembrane pressure (TMP) was integral to water permeation, while the molecular weight of contaminants (*C*_Da), concentration of contaminants (*C*_mg/L), and concentration of foulants (*F*_mg/L) played crucial roles in removal efficiency- and membrane fouling-related performance. As demonstrated in Figure 3a, TMP was negatively correlated with water permeation. Such a negative effect of applied hydraulic pressure on water flux was mainly ascribed to the compaction of the polymeric membrane.^55,56^ The compaction under different applied pressures resulted in a reduction in the membrane pore size and porosity, which agreed well with the contribution of pore size and porosity to water permeability (Figure 3b). *C*_Da was positively correlated with removal efficiency (Figure S5a). The separation of UF membranes was dictated by the molecular-sieving mechanism indicated by the molecular weight cutoff (MWCO), where the large solutes were retained by the smaller pores to achieve high removal efficiency.^57^ The negative correlation of the mean pore radius with removal efficiency in Figure S6a is consistent with this domain knowledge. These findings verified the necessity of developing an ML model with operational conditions included (in addition to the fabrication parameters) as input features.

**Figure 4:**
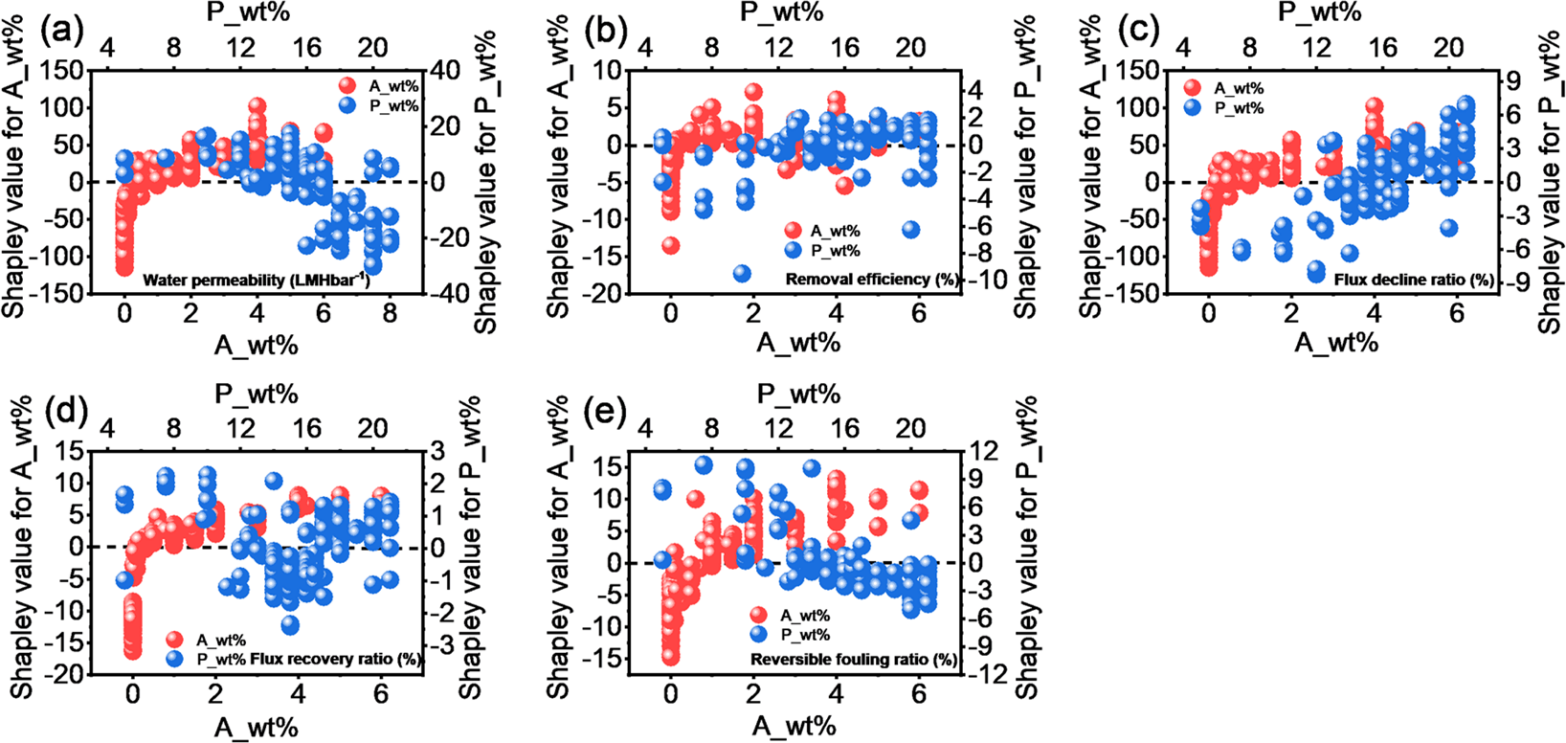
Shapley values of additive loading (*A*_wt %) and polymer content (*P*_wt %) for each of the membrane performance indices: (a) water permeability, (b) removal efficiency, (c) flux decline ratio, (d) flux recovery ratio, and (e) reversible fouling ratio.

As shown in Figure 4, *A*_wt % was positively correlated with water permeability, removal efficiency, and flux recovery ratio with a loading of >0.5 wt % (defined by the weight percentage of an additive to the base polymer), while the beneficial loading for the flux decline ratio and reversible fouling ratio was found to be >1.0 wt % (Figure 4c,e). These findings revealed that incorporating 1.0 wt % additives (specifically nanomaterials) into a polymer matrix could potentially afford a membrane with high water permeability and removal efficiency, as well as antifouling performance. As the backbone of a membrane, the polymer used to fabricate the UF membrane was expected to be a crucial feature. As shown in Figure 4, *P*_wt % had a significant impact on membrane performance, especially on water permeability. With *P*_wt % ranging from 10 to 16 wt %, it was positively correlated with water permeability. Beyond 16 wt %, a strongly negative correlation with water permeability was observed. It might be attributed to the delayed phase inversion due to the higher polymer content, which normally resulted in less porosity and a small pore sizes in the membrane.^58^ This trend was in accordance with the results shown in Figure 3b, wherein the pore size and porosity exhibited a positive correlation with water permeability. Notably, as for removal efficiency, flux recovery ratio, and reversible fouling ratio, the Shapley value of *P*_wt % was comparable to that of *A*_wt %, while in terms of water permeability and flux decline ratio, the Shapley value of *A*_wt % was much larger than that of *P*_wt %, suggesting that in comparison with tuning the polymer content, tailoring the addition loading of nanomaterials into the polymer matrix might be a more effective method for achieving a UF membrane with desirable membrane performance, especially enhanced water permeability and a decreased flux decline ratio. In practical water and wastewater treatment applications, UF membranes with a lower flux decline ratio are desired as it indicates that such a membrane holds better antifouling potential.

**Figure 5:**
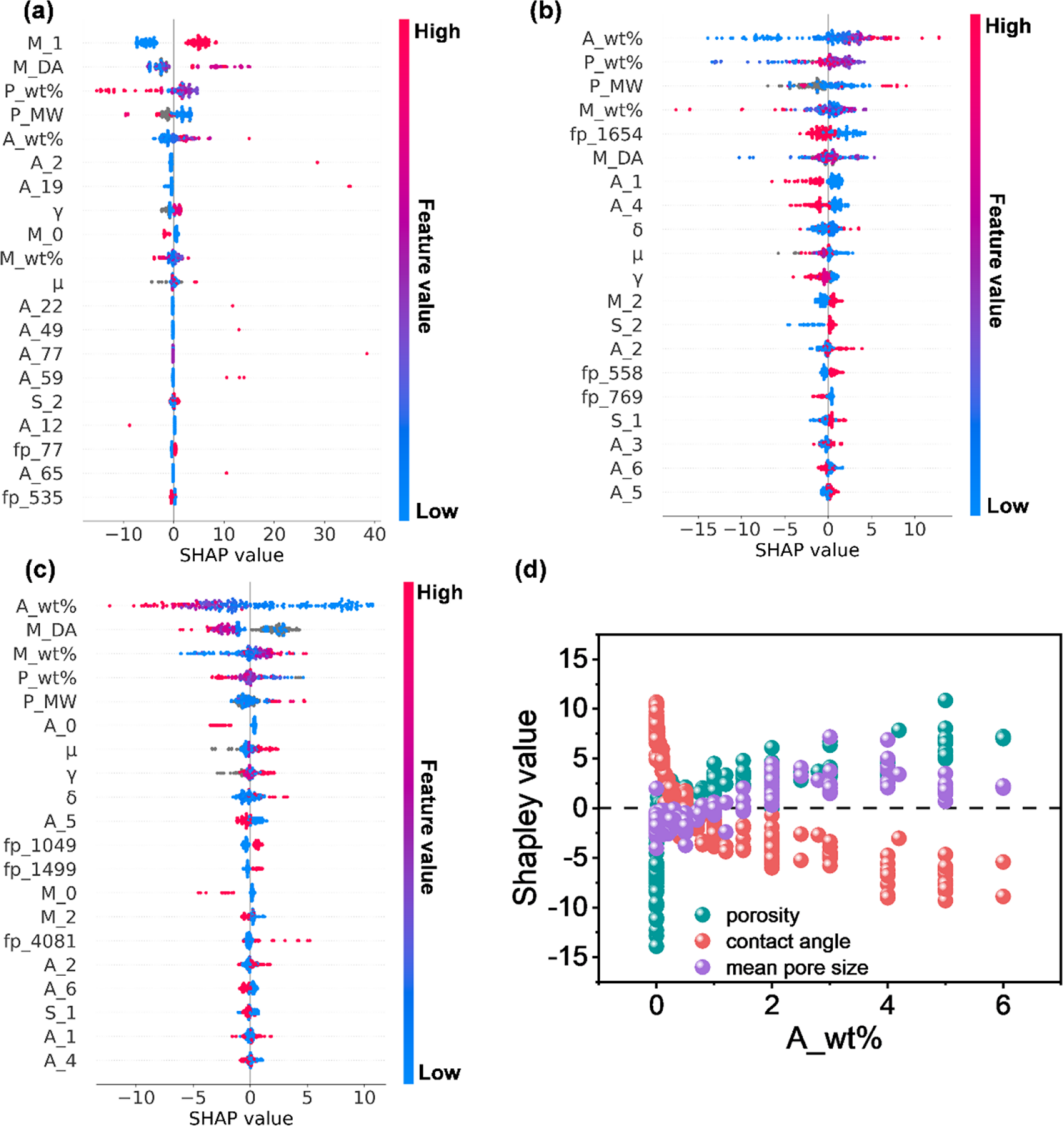
SHAP plot for ML models on membrane properties: (a) mean pore size, (b) overall porosity, (c) contact angle, and (d) Shapley values of *A*_wt % for each of the membrane properties. *A*_number (e.g., *A*_2) denotes the encoder for the category feature (i.e., the type of additive). The feature number (e.g., fp_1654) denotes the feature position in the Morgan fingerprint vector. The chemical structure of fp_1654 is shown in Figure S7.

Figure 5 displays the feature analysis of the ML models for the prediction of membrane properties. The ranking of the features’ importance for predicting membrane properties was in accordance with that for membrane performance prediction. In the analogue to the prediction of membrane performance, *A*_wt %, *P*_wt %, *M*_Da, and *M*_wt % were also found to be the four most significant fabrication factors dominating membrane property prediction. Therefore, the influence of these factors on membrane performance prediction can be explained by their contributions to each of the membrane properties. *A*_wt % was positively correlated with the mean pore size and overall porosity and negatively correlated with the contact angle at a loading of >1.0 wt % (Figure 5d), which was highly consistent with the beneficial range of *A*_wt % for membrane performance. This revealed that an increased addition of additives (referring to nanomaterials in the collected data sets) contributed to the formation of a larger pore size, a higher porosity, and a smaller contact angle indicating higher surface hydrophilicity,^59,60^ typically leading to desirable water permeability,^61,62^ which agreed well with the results shown in Figure 3b.

It has been reported that irreversible fouling rapidly occurred as a result of internal pore blockage, followed by the formation of a cake layer on the membrane surface.^63^ The pore constriction-induced irreversible fouling resulted in the progressive decline of the membrane water flux. Therefore, a smaller pore might make it difficult for foulants to enter and constrict the pores, which in turn could afford a lower flux decline ratio. As shown in Figure S6, the mean pore radius positively correlated with the flux decline ratio, revealing that a smaller mean pore radius contributed to a lower flux decline ratio, which was a desirable performance for separation membranes. *P*_wt %, *A*_wt %, *P*_MW, and *M*_Da were found to be the four most influential factors in predicting the flux decline ratio (Figure S5b). A possible reason could be these four factors showed significant effects on the mean pore size of the membrane as illustrated in Figure 5a. The flux recovery ratio and reversible fouling ratio, which could be promoted through hydraulic cleaning, largely depended on the membrane surface hydrophilicity inferred from the contact angle. The ranking of features for the flux recovery ratio and the reversible fouling ratio indicated that the contact angle was the most important property for both performance indices. The four most important factors governing the prediction of the flux recovery ratio and the reversible fouling ratio were identified as *A*_wt %, *P*_wt %, *M*_Da, and *M*_wt %, which was consistent with the top four factors contributing to the contact angle.

## Supporting Information Available

Reagents and materials (Text S1), ultrafiltration membrane fabrication (Text S2), input features and target used in the data sets (Table S1), screening candidates for encoder methods (Table S2), percentage of the missing value for each feature (Table S3), predictive performance of different configurations of machine learning algorithms and encoder methods for the water permeability data set (Table S4), range of candidate hyperparameters for each ML algorithm (Table S5), detailed conditions for fabricating and testing UF membranes (Table S6), predictive performance of the ML models with membrane properties included as input features (Table S7), predicted and experimental results of the fabricated membranes (Table S8), predicted and characterized membrane properties of the fabricated membranes (Table S9), data distribution of the numeric input features (Figure S1), types of additives involved in the UF membrane as collected from the literature (Figure S2), Pearson correlation coefficients (PCCs) (Figure S3), plot of training RMSE (RMSEcv-training, Loss) versus validation RMSE (RMSEcv-validation, Train_loss) (Figure S4), SHAP plot for ML models (Figure S5), SHAP plot for ML models accounting for membrane properties (Figure S6), and chemical structure of feature fp_1654 (Figure S7) (PDF)
Supplementary data sets (XLSX)

## Accession Codes

Codes related to this paper can be found in Github (https://github.com/Shifa-Zhong/UF).
