# Supplementary material for: Understanding and Designing a High-Performance Ultrafiltration Membrane Using Machine Learning
Source: Environ Sci Technol. 2023 Feb 15;57(46):17831–40. doi: 10.1021/acs.est.2c05404 (PMC10666290; doi:10.1021/acs.est.2c05404)
Supplement: Supplementary file 1 — es2c05404_si_001.pdf [file es2c05404_si_001.pdf]

Supporting Information

Understanding and designing a high-performance ultrafiltration membrane using machine learning

Haiping Gao,[a] Shifa Zhong,[a,b] Raghav Dangayach,[a] Yongsheng Chen[a,*]

[a]School of Civil and Environmental Engineering, Georgia Institute of Technology, Atlanta, Georgia 30332, United States

[b]School of Ecological and Environmental Sciences, East China Normal University, Shanghai 200241, China

[*]Corresponding author: Yongsheng Chen, Email: yongsheng.chen@ce.gatech.edu, Phone: +14048943089

This supporting information includes:

Number of pages (including the cover page): 12

Number of figures: 7

Number of tables: 9

Text S1: Reagents and Materials (Page S2)

Text S2: Ultrafiltration membrane fabrication (Page S2)

Text S1 Reagents and Materials

PVDF powder (534,000 g mol^{-1} and 275,000 g mol^{-1}), PVC powder (), polyvinylpyrrolidone (PVP, 40 kDa), poly(ethylene glycol) (PEG, 2000 Da and 6000 Da), dimethylformamide (DMF, ≥99%), 1-Methyl-2-pyrrolidinone (NMP, anhydrous, ≥99.5%), bovine serum albumin (BSA, 66 kDa), and humic acid sodium salt (HA, technical grade) were all purchased from Sigma Aldrich and used as received without further purification.

Text S2 Ultrafiltration membrane fabrication

Three ultrafiltration membranes were prepared using the nonsolvent-induced phase inversion (NIPS) method. In brief, the homogenous casting solutions were prepared by dissolving certain amount of polymer and pore maker in organic solvent at elevated temperature of 60 ℃ under moderate stirring. For membranes with incorporation of nanomaterials, the nanomaterial was first dispersed into the organic solvent and sonicated for 2h to obtain the suspension solution of nanomaterial. Subsequently, the polymer and pore maker were added into the above solution for casting solution preparation. The homogenous casting solution was cooled to room temperature and degassed in a vacuum oven at ambient temperature for 1h. Then, the prepared solution was casted onto a glass plate using a Dr. Blade at a gap height of 200 µm. After that, the casted solution with the glass plate was immediately immersed in a water (fresh DI water) coagulation bath at room temperature to trigger the phase inversion. Detailed membrane fabrication conditions were summarized in Table S6.

Table S1 The input features and targets used in the data sets.

Input features	Unit	Description of input features
**Polymer**		
P (SMILES)	/	The base polymer used to fabricate membranes.
P_MW	g mol^{-1}	The molecular weight of base polymer.
δ	mN m^{-1}	The surface tension of base polymer.
**Fabrication condition**		
P_wt%	wt%	The weight fraction of base polymer in the casting solution used to fabricate membranes.
M	/	The pore maker used in the casting solution.
M_wt%	wt%	The weight fraction of pore maker in the casting solution.
M_Da	Da	The molecular weight of pore maker.
A	/	The additive type blended into the polymer matrix.
A_wt%	wt%	The weight fraction of incorporated additive to the base polymer.
S	/	The organic solvent used to prepare casting solution.
γ	$MPa^{1/2}$	The solubility parameter of the organic solvent used.
**Membrane property**		
Mean pore radius	nm	The mean pore size of the fabricated membrane.
Overall porosity	%	The overall porosity of the fabricated membrane.
Contact angle	degree	The static contact angle of the fabricated membrane.
Surface roughness	nm	The root mean square roughness of the UF membrane.
**Operational condition**		
TMP	bar	The applied hydraulic pressure through membrane performance test.
C	/	The contaminants used for removal efficiency measurement.
C_Da	Da	The molecular weight of contaminants.
C_mg/L	mg L^{-1}	The initial concentration of contaminants in the feed solution.
F	/	The model foulant used for membrane fouling evaluation.
F_mg/L	mg L^{-1}	The initial concentration of model foulant in the feed solution.
**Target (Membrane performance)**		
Water permeability	LMH bar^{-1}	The pure water permeability of the UF membrane.

Removal efficiency	%	The rejection rate towards the contaminants.
Flux decline ratio	%	The flux decline ratio when using solution containing contaminants as feed solution.
Flux recovery ratio	%	The flux recovery ratio after physical cleaning.
Reversible fouling ratio	%	The reversible fouling ratio of the membrane.

Table S2 Screening candidates for Encoder methods.

Methods	Candidates	Working principle
Encoding methods	BackwardDifferenceEncoder	The mean of the dependent variable for a level is compared with the mean of the dependent variable for the prior level.
	MEstimateEncoder,	M-Estimate Encoder is a simplified version of Target Encoder. It has only one hyper-parameter — m, which represents the power of regularization.
	SumEncoder	Sum Encoder compares the mean of the dependent variable (target) for a given level of a categorical column to the overall mean of the target
	BinaryEncoder	Converting a category into binary digits. If there are n unique categories, then binary encoding results in the only log(base 2)^n features
	JamesSteinEncoder	For feature value, the James-Stein estimator returns a weighted average of: The mean target value for the observed feature value or the mean target value (regardless of the feature value).
	OneHotEncoder	Mapping each category to a vector that contains 0 and 1.
	BaseNEncoder	Base-N encoder encodes the categories into arrays of their base-N representation. A base of 1 is equivalent to one-hot encoding (not really base-1, but useful), a base of 2 is equivalent to binary encoding. N=number of actual categories is equivalent to vanilla ordinal encoding.
	HelmertEncoder	The mean of the dependent variable for a level is compared to the mean of the dependent variable over all previous levels

Table S3 Percentage of the missing value for each feature.

Feature	Data size	Percentage of Missing value (%)
Polymer type	320	0.0
P_MW	200	37.4
P_wt%	320	0.0
Pore maker type	320	0.0
M_Da	220	31.1

M_wt%	320	0.0
Additive type	320	0.0
A_wt%	320	0.0
Organic solvent type	320	0.0
Solubility parameter	315	1.6
TMP	320	0.0
Organic compound	320	0.0
C_mg/L	320	0.0
C_Da	258	19.4
Foulant type	320	0.0
F_mg/L	320	0.0
Water permeability	320	0.0
Removal efficiency	320	0.0
Flux decline ratio	320	0.0
Flux recovery ratio	320	0.0
Reversible fouling ratio	320	0.0
Irreversible fouling ratio	320	0.0

Table S4 Predictive performance of different configurations of machine learning algorithms and encoder methods for the water permeability data set.

**Dataset**	**R^2 (training)**	**RMSE (training)**	**R^2 (test)**	**RMSE (test)**	**Regressor**	**Encoder**
Water permeability	0.9937	17.3547	0.8725	67.9276	CatBoostRegressor	BaseNEncoder
	0.9937	17.3547	0.8725	67.9276	CatBoostRegressor	BinaryEncoder
	0.9968	14.2858	0.8838	71.3299	XGBRegressor	OneHotEncoder
	0.9902	20.0295	0.8367	74.3563	CatBoostRegressor	BackwardDifferenceEncoder
	0.9815	25.2447	0.8817	76.0850	CatBoostRegressor	OneHotEncoder
	0.9974	14.2803	0.8711	77.5317	XGBRegressor	HelmertEncoder
	0.9978	14.5355	0.8394	78.3169	XGBRegressor	BaseNEncoder
	0.9978	14.5355	0.8394	78.3169	XGBRegressor	BinaryEncoder
	0.9968	14.2858	0.8825	78.3911	XGBRegressor	SumEncoder
	0.9978	14.5117	0.8599	81.0735	XGBRegressor	BackwardDifferenceEncoder

0.9864	21.7942	0.8742	84.4263	CatBoostRegressor	HelmertEncoder
0.9825	23.9601	0.8235	103.4916	CatBoostRegressor	SumEncoder
0.9925	17.4184	0.8438	106.1097	CatBoostRegressor	MEstimateEncoder
0.9921	17.8510	0.8299	108.8766	CatBoostRegressor	JamesSteinEncoder
0.9978	14.4793	0.8013	111.5431	XGBRegressor	MEstimateEncoder
0.9978	14.4221	0.8144	117.9311	XGBRegressor	JamesSteinEncoder

Table S5 The range of candidate hyperparameters for each ML algorithm.

ML algorithm	Hyperparameters	Range
CatBoost	depth	1-6
	L2_leaf_reg	3-100
	Learning_rate	0.0001-0.025
	iterations	1-1000
	Bagging temperature	1-200
	Random strength	1-200
XGBoost	Colsample_bytree	0-1
	Learning rate	0-1
	Max_depth	1-6
	subsample	0-1
	Reg_alpha	0-10
	gamma	0-20
	Reg_lambda	1-10
	N_estimators	1-100
fingerprint	Fp_radius	0-5
	Fp_length	10-5048

Table S6 The detailed conditions for fabricating and testing UF membranes.

Conditions	Membrane #1	Membrane #2	Membrane #3
Polymer	Polyvinylidene fluoride	Polyvinylidene fluoride	Polyvinyl chloride

P_MW (g mol^{-1})	534000	275000	62000
P_wt%	15	16	13.5
δ (mN m^{-1})	29.2	29.2	40
Pore maker	PEG	PEG	PVP
M_Da	2000	6000	40000
M_wt%	3	6	5
Additive	none	TiO2	OMWCNT
A_wt%	0	0.02	0.01
Organic solvent	NMP	DMF	NMP
γ (MPa$^{1/2}$)	22.9	24.8	22.9
TMP (bar)	1.5	1	1
Contaminant	BSA	BSA	BSA
C_Da	66000	66000	66000
C_mg/L	150	100	50
Foulant	HA	HA	HA
F_mg/L	20	20	20

Table S7 Predictive performance of the ML models with membrane properties included as input features.

Performance indices	Unit	Total data size	Training set R^2	Training set RMSE	Test set R^2	Test RMSE
Water permeability	LMHbar-1	320	0.98	24.29	0.78	74.90
Removal efficiency	%	320	0.98	2.33	0.88	6.69
Flux decline ratio	%	320	0.97	3.38	0.80	9.01
Flux recovery ratio	%	320	0.95	2.89	0.56	8.68

Reversible fouling ratio	%	320	0.98	2.58	0.76	9.58

Table S8 Predicted and experimental results of the fabricated membranes.

Membrane performance	Unit	Membrane #1		Membrane #2		Membrane #3	
		E[a]	P[b]	E	P	E	P
Water permeability	LMHbar^{-1}	121.03	109.12	173.26	164.85	205.30	181.01
Removal efficiency	%	92.71	87.24	88.27	86.95	85.77	86.45
Flux decline ratio	%	50.18	43.81	52.03	40.69	54.32	49.55
Flux recovery ratio	%	55.83	59.29	71.56	67.15	65.37	61.58
Reversible fouling ratio	%	17.52	14.75	20.11	14.35	15.86	10.53

[a] Experimental results; [b] Predicted values

Table S9 Predicted and characterized membrane properties of the fabricated membranes.

Membrane properties	Unit	Membrane #1		Membrane #2		Membrane #3	
		E[a]	P[b]	E	P	E	P
Overall porosity	%	60.83	68.19	67.27	70.16	60.32	53.74
Mean pore radius	µm	14.21	12.84	15.67	16.96	30.11	34.86
Contact angle	degree	68.03	78.06	60.51	74.45	66.25	77.36

[a] Characterized results; [b] Predicted values

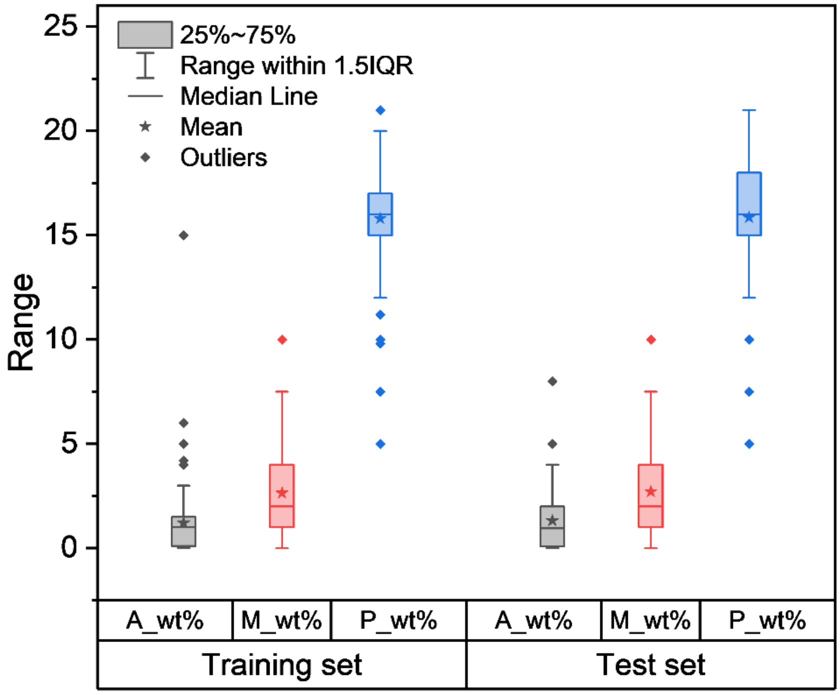

Figure S1. Data distribution of the numeric input features.

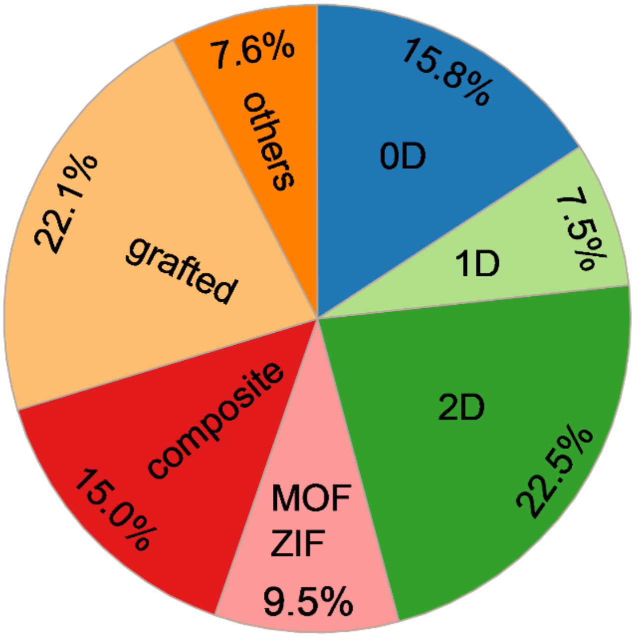

Figure S2. Types of additives involved in UF membrane as collected from the literature.

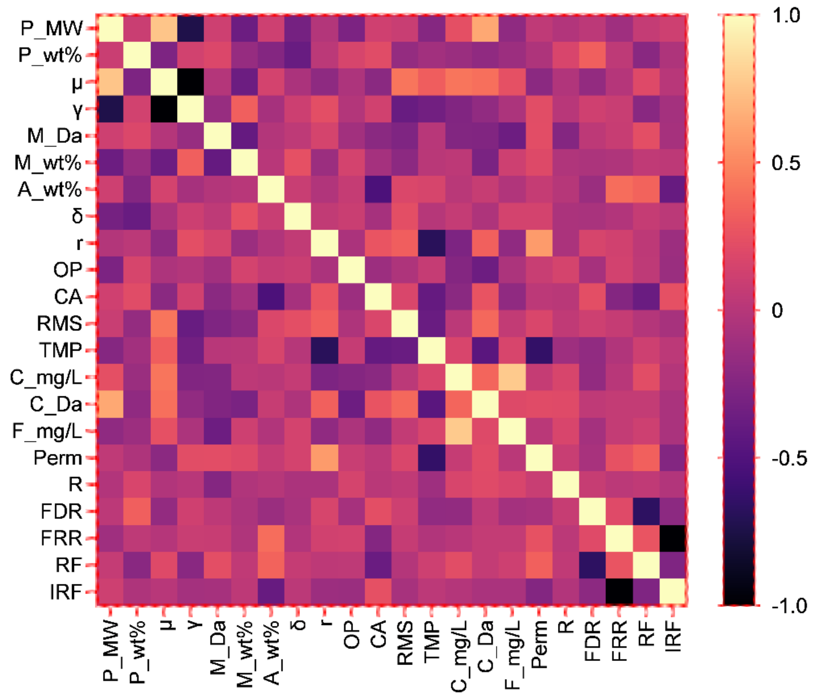

Figure S3. Pearson correlation coefficients (PCCs).

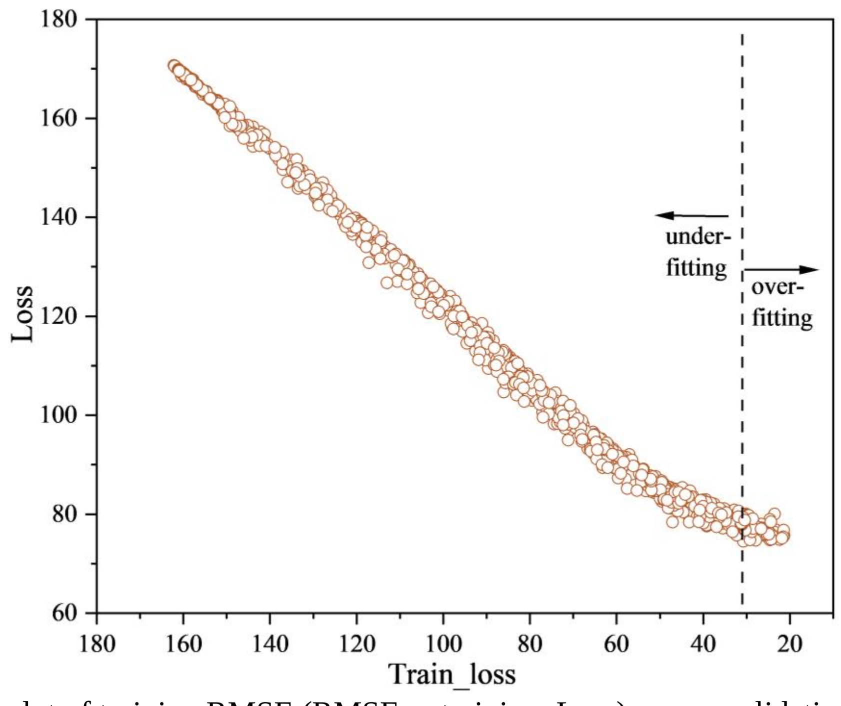

Figure S4. The plot of training RMSE (RMSEcv-training, Loss) versus validation RMSE (RMSEcv-validation, Train_loss).

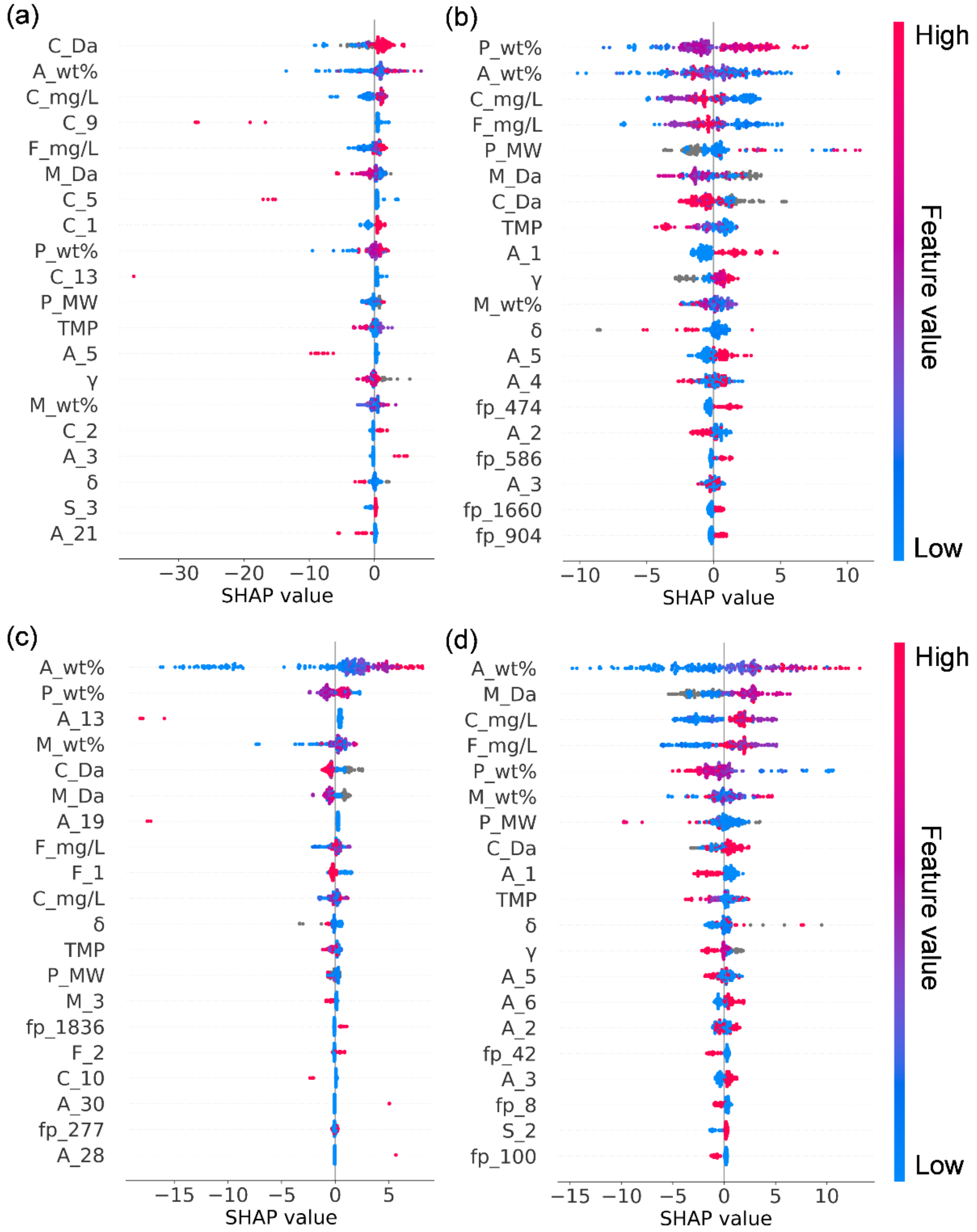

Figure S5. The SHAP plot for ML models. (a) Removal efficiency (%). (b) Flux decline ratio (%). (c) Flux recovery ratio (%). (d) Reversible fouling ratio (%). Feature number (e.g., fp_474) denotes the feature position in the Morgan fingerprint vector.

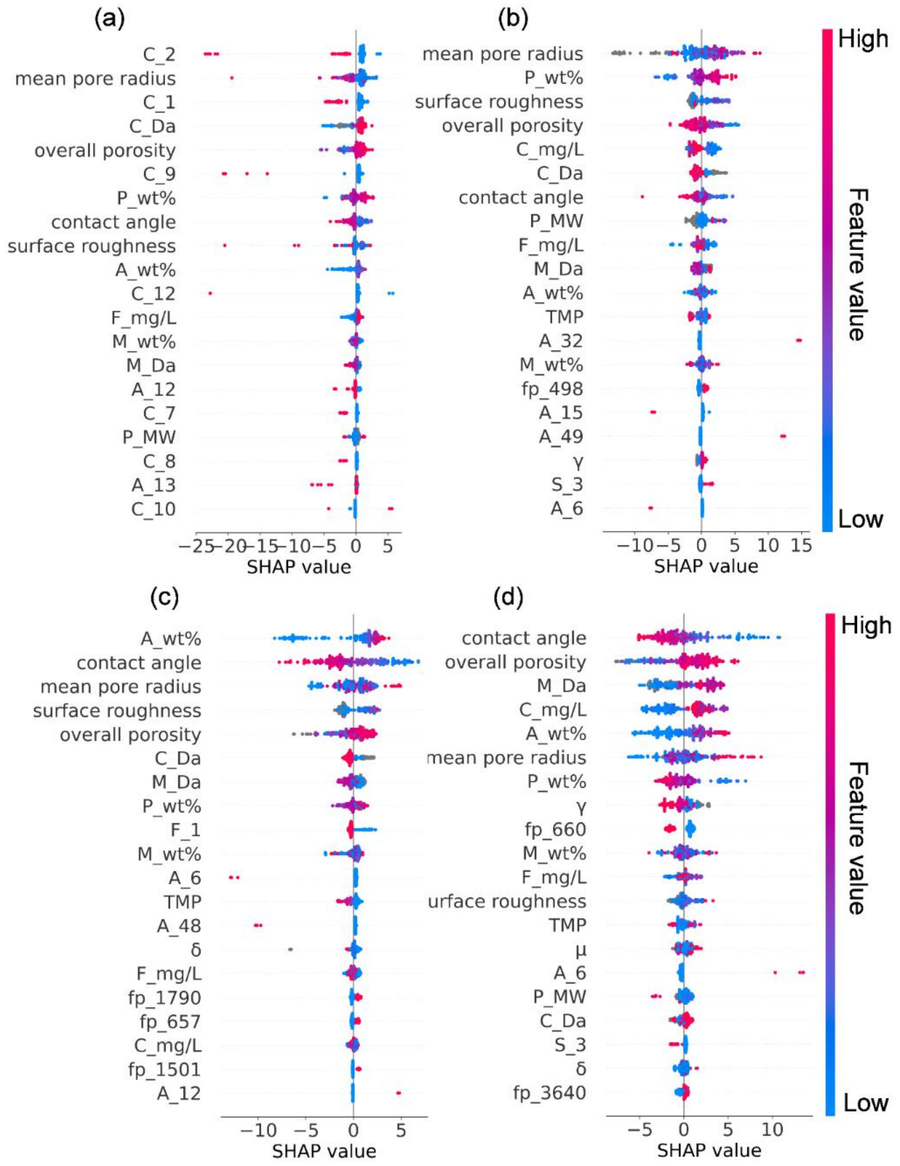

Figure S6. The SHAP plot for ML models accounting for membrane properties. (a) Removal efficiency. (b) Flux decline ratio. (c) Flux recovery ratio. (d) Reversible fouling ratio. Feature number (e.g., fp_498) denotes the feature position in the Morgan fingerprint vector.

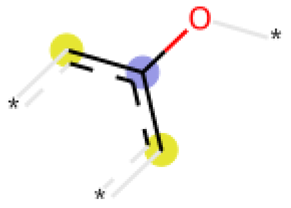

Figure S7. The chemical structure of feature fp_1654.
